# Using machine-learning models to predict extubation failure in neonates with bronchopulmonary dysplasia

**DOI:** 10.1186/s12890-024-03133-3

**Published:** 2024-07-01

**Authors:** Yue Tao, Xin Ding, Wan-liang Guo

**Affiliations:** 1 Department of radiology, Children’s Hospital of Soochow University, 92 Zhongnan District, Suzhou, Jiangsu 215025 China; 2 Department of neonatology, Children’s Hospital of Soochow University, 92 Zhongnan District, Suzhou, Jiangsu 215025 China

**Keywords:** Brochopulmonary dysplasia, Neonates, Machine learning, Extubation failure, Extreme gradient boosting (XGBoost)

## Abstract

**Aim:**

To develop a decision-support tool for predicting extubation failure (EF) in neonates with bronchopulmonary dysplasia (BPD) using a set of machine-learning algorithms.

**Methods:**

A dataset of 284 BPD neonates on mechanical ventilation was used to develop predictive models via machine-learning algorithms, including extreme gradient boosting (XGBoost), random forest, support vector machine, naïve Bayes, logistic regression, and k-nearest neighbor. The top three models were assessed by the area under the receiver operating characteristic curve (AUC), and their performance was tested by decision curve analysis (DCA). Confusion matrix was used to show the high performance of the best model. The importance matrix plot and SHapley Additive exPlanations values were calculated to evaluate the feature importance and visualize the results. The nomogram and clinical impact curves were used to validate the final model.

**Results:**

According to the AUC values and DCA results, the XGboost model performed best (AUC = 0.873, sensitivity = 0.896, specificity = 0.838). The nomogram and clinical impact curve verified that the XGBoost model possessed a significant predictive value. The following were predictive factors for EF: pO_2_, hemoglobin, mechanical ventilation (MV) rate, pH, Apgar score at 5 min, FiO_2_, C-reactive protein, Apgar score at 1 min, red blood cell count, PIP, gestational age, highest FiO_2_ at the first 24 h, heart rate, birth weight, pCO_2_. Further, pO_2_, hemoglobin, and MV rate were the three most important factors for predicting EF.

**Conclusions:**

The present study indicated that the XGBoost model was significant in predicting EF in BPD neonates with mechanical ventilation, which is helpful in determining the right extubation time among neonates with BPD to reduce the occurrence of complications.

## Introduction

Bronchopulmonary dysplasia (BPD) is one of the most common and serious complications in preterm infants. The diagnosis is based on the recommended criterion, that is, any newborn with oxygen dependence (fraction of inspired oxygen [FiO_2_] > 0.21) for ⩾ 28 days) [1]. Extubation failure (EF) is a difficult problem in newborns and neonates diagnosed with BPD, and it could result in poorer prognosis, such as pneumothorax, subglottic injury, nosocomial infection, and neurodevelopmental impairment [2]. EF is defined as the need for re-intubation within 72 h after planned extubation [3], and timely and effective extubation is an important goal [4]. However, premature extubation in unprepared neonates causes EF, prolonged mechanical ventilation (MV) duration, and increased morbidity and mortality in high-risk neonates [5–7]. A variety of prospective and retrospective studies have tried to identify predictors of EF, and different factors predicting EF have been studied, including birth weight, Apgar score [8], FiO_2_ prior to extubation [9], spontaneous breathing tests [10], cardiorespiratory variability [11], airway trauma, and feeding difficulties [12].

With the rapid development of precision medicine, novel machine-learning (ML) techniques have offered improved predictive performance relative to traditional prediction methods [13]. A previous study based on 486 premature neonates developed different ML algorithms to predict EF, but the algorithms performed worse than expert clinicians [14]. Kanbar et al. [15] mainly applied a single classifier to predict EF among 241 extremely preterm infants in a multicenter study. The authors obtained the receiver operating characteristic (ROC) curves of clinical, cardiorespiratory, and clinical and cardiorespiratory classifiers. The performance of their models was unsatisfactory (all area under the ROC curve [AUC] values < 0.75), and they failed to further explore visualization results. Onu et al. [16] intended to model the sequence of respiratory patterns for the 5-minute period of Endo Tracheal Tube Continuous Positive Airway Pressure (ETT-CPAP) and use a Markov chain model to convert state information into probabilities and predict the probability of EF in extremely preterm newborns. Robles-Rubio et al. [17] used an automated analysis of Respiratory Inductive Plethysmography (RIP) signals in a limited number of infants, which was a simple classification using two metrics and was a hypothesis generator. However, the pathophysiological relevance of the metrics derived from an Automated Unsupervised Respiratory Event Analysis (AUREA) remains unknown.

Extreme gradient boosting (XGBoost) is an interpretable model that has been used in different disease models, including in neonates [18–21]. However, there have been no reports on the use of XGBoost to study EF. In this study, we proposed an EF prediction and feature analysis model using XGBoost. The results indicated that the prediction model was helpful in increasing the current successful extubation rate in neonates under MV.

## Materials and methods

### Selection of participants

The study was approved by the Ethics Committee of the Children’s Hospital of Soochow University, with a waiver for informed consent because of the retrospective nature of the study. Eligible neonates were those who had been mechanically ventilated due to a diagnosis of BPD between January 1, 2015, and May 31, 2022, and survived to their first extubation from MV. The study excluded neonates (1) who were never mechanically ventilated or died prior to their first extubation, (2) who experienced unplanned extubation episodes and required immediate re-intubation without a trial of noninvasive respiratory support, (3) who were diagnosed with severe congenital malformations, complex congenital heart disease, chromosomal abnormalities, genetic metabolism sexual diseases, severe infectious diseases; and (4) who were missing too many medical records.

BPD was diagnosed based on the recommended diagnostic criterion, that is, any newborn with oxygen dependence (FiO_2_ > 0.21) for ⩾ 28 days). If a newborn’s gestational age (GA) was less than 32 weeks, BPD was graded according to the corrected GA of 36 weeks or oxygen concentration at discharge. If the GA was more than 32 weeks, BPD was graded according to the oxygen demand concentration at 56 days after birth or at discharge as follows: (1) mild, no oxygen; (2) moderate, FiO_2_ < 0.30; and (3) severe, FiO_2_ < 0.30 or requiring mechanical ventilation [1].

EF was defined as the need for re-intubation within 72 h after planned extubation if blood gas values, vital signs, saturation, and overall clinical stability of the neonate were abnormal, according to the commonly used definition [3]. The criteria for extubation in the present study were as follows: (1) The neonates’ primary disease improved, infection was controlled, and they were in good condition. (2) When the blood gas analysis was normal, the ventilator parameters were gradually reduced, and spontaneous breathing was exercised and enhanced. We reduced FiO_2_ and PIP first; then, the MV rate was reduced, chest movements were observed, and SaO_2_ and arterial blood gas results were monitored. (3) When PIP was ≤ 18 cm H_2_O, PEEP was 2–4 cm H2O, rate was ≤ 10 times/min, FiO_2_ was ≤ 0.4, and the arterial blood gas results were normal, the MV was removed. The criteria for re-intubation in the present study were as follows: (1) symptoms of dyspnea persisted during treatment after extubation; (2) the arterial pCO_2_ was above 70 mm Hg; and (3) the arterial pO_2_ was below 50 mm Hg [3].

All of the neonates in this study received post-extubation noninvasive respiratory support, and only the first extubation episode was evaluated.

### Data collection

Clinical and laboratory variables, vital signs, and MV parameters were obtained within 6 h prior to extubation. The recorded characteristics included sex, birth weight (BW), GA, delivery mode, Apgar scores at 1 min and 5 min, delivery room (DR) resuscitation (chest compression, epinephrine), multiple-pregnancy status, intrauterine distress, prenatal maternal use of dexamethasone and antibiotics, placental abnormality, premature rupture of membranes, diseases during pregnancy (including pregnancy-induced hypertension and gestational diabetes), highest FiO_2_ at the first 24 h, laboratory data (arterial blood gas, full blood count, and C-reactive protein [CRP]), vital signs (respiratory rate and heart rate), and MV parameters (PIP, FiO_2_, PEEP, and MV rate). For variables with multiple measurements, the average values were assessed.

### Statistical analysis

The baseline characteristics were compared between the successful-extubation group and the EF group. Continuous variables without normal distribution were reported as medians (interquartile ranges) and analyzed using the Wilcoxon rank-sum test, while continuous variables with normal distribution were presented as means (standard deviations) and analyzed using the Student’s t test. Categorical variables were reported as numbers and analyzed using the chi-square test, continuity-corrected chi-square test, or Fisher’s exact-probability method for intergroup comparisons. All of the statistical analyses were conducted using Python (version 3.6) and R software. P values lower than 0.05 were defined as being statistically significant.

### Prediction models

To address the issue of imbalanced data, the oversampling method with Adaptive Synthetic Sampling (ADASYN) algorithm was utilized. Unlike other oversampling techniques, ADASYN generates synthetic samples based on the density between different samples to achieve a balanced class distribution. We used a dynamic K value approach to more accurately capture the data characteristics of each class. Initially, each class sample was assigned a starting K value of 5. Local density was then calculated by assessing the proportion of majority class samples among the K nearest neighbors, estimating the sample’s regional density. K values were dynamically adjusted based on this density. Lower density samples received higher K values to better represent their data distribution, while higher density samples got smaller K values due to denser surrounding data. Synthetic samples were generated using these adjusted K values, particularly for minority class samples, selecting from their K nearest neighbors to enhance quality and diversity [22]. Six ML methods, namely, K-nearest neighbor (KNN), support vector machine (SVM), logistic regression (LR), naïve Bayes (NB), random forest (RF), and XGBoost, were preliminarily used to predict EF. Ten-fold cross validation was conducted in the complete dataset and the average metrics values were exported to access model performance in current study. During the process of hyperparameters tuning, the models were initialized separately. Afterwards, batch gradient descent algorithm was applied iteratively to update these parameters until convergence is achieved. In each iteration, the batch gradient descent algorithm adjusts the values of parameters based on the gradient of the loss function, and then gradually stabilizing the loss function values of these models. While the XGBoost algorithm can handle missing values automatically, most of the other models cannot analyze data with missing values. Therefore, features with missing degrees and other missing values were imputed using multiple imputation. In the model-comparison phase, the performances of the six predictive models were tested by the AUC values and compared, and decision curve analysis (DCA) was used to select the optimal model that achieved the highest overall diagnostic value for further verification. Confusion matrix was used to show the high performance of the best model. Feature importance analysis was performed to identify the top 15 features that were helpful for EF prediction. SHapley Additive exPlanations (SHAP)—an interpretable tool for predicting the output of ML models—was used to reflect the influence of the features in each data sample and also show positive and negative effects [23]. Finally, the nomogram and clinical impact curve (CIC) were plotted to evaluate the clinical usefulness and applicability of the model with the best diagnostic value.

## Results

### Characteristics affecting EF in BPD neonates

**Fig. 1:**
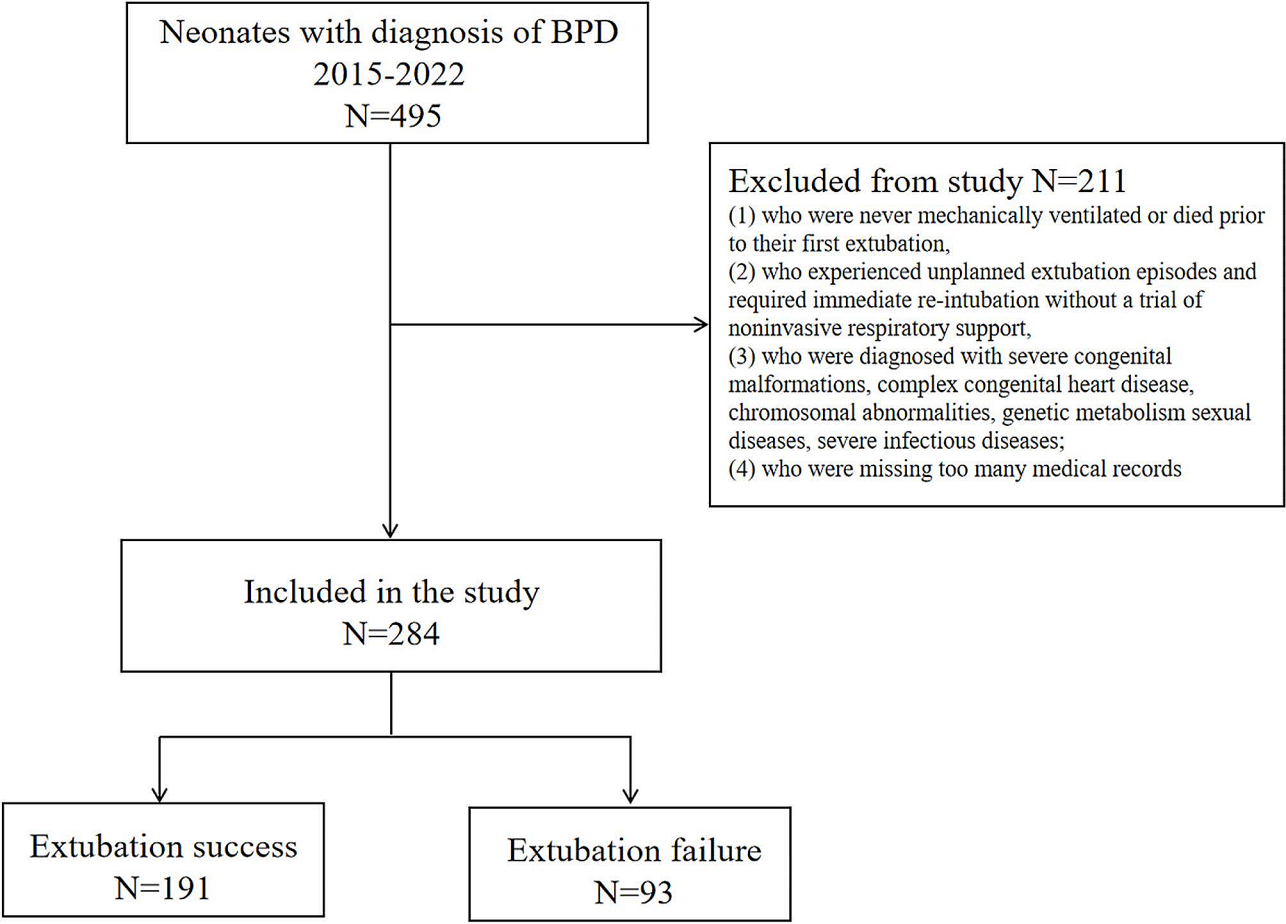
Flowchart of the study

As shown in Fig. 1, a total of 284 neonates were included in this cohort. The dataset was then divided into a success set (*n* = 191) and a failure set (*n* = 93). Comparisons of the baseline characteristics, vital signs, laboratory parameters, and MV parameters before extubation between the successful-extubation and EF groups are shown in Table 1. There were significant differences in BW, GA, Apgar scores at 1 min and 5 min, DR chest compression, use of PS and respiratory stimulant, maternal disease (pregnancy-induced hypertension), and highest FiO_2_ in the first 24 h between the two groups (*P* < 0.05). The neonates in the EF group had a higher heart rate, MV rate, FiO_2_, PEEP, PIP, abnormal arterial blood gas (pH, pO_2_, and pCO_2_), and full blood count (red blood count [RBC], hemoglobin [Hb]) within 6 h prior to extubation (*P* < 0.05). Significantly prolonged MV duration, younger age at extubation, and higher CRP levels within 6 h before extubation were also found in the EF group. However, sex, premature rupture of membranes, intrauterine distress, abnormal placenta, delivery mode, multiple pregnancy, maternal disease (such as gestational diabetes), DR epinephrine, PMA at extubation, PLT, and WBC were not significantly different between the two groups.

**Table 1:** Baseline characteristics between the successful extubation group and the EF group

	Success(*n* = 191)	Failure(*n* = 93)	*P*-value
Demographics			
Gender			0.422
Male n (%)	118(61.8)	62(66.7)	
Female n (%)	73(38.2)	31(33.3)	
GA (wk) median [Q1,Q3]	29.3(27.8, 30.8)	28.5(26.3, 30.4)	0.014
BW (g) median [Q1,Q3]	1215(1080, 1500)	1100(900, 1395)	0.001
Premature rupture of membranes n (%)	60(31.4)	21(22.6)	0.122
Intrauterine distress n (%)	12(6.3)	10(10.8)	0.186
Abnormal placenta n (%)	26(13.6)	15(16.5)	0.571
Caesarean delivery n (%)	90(47.1)	41(45.1)	0.63
Multiple pregnancy n (%)	31(34.1)	18(19.8)	0.513
Maternal disease n (%)			
pregnancy-induced hypertension n (%)	20(6.9)	19(20.9)	0.022
gestational diabetes n (%)	8(4.2)	3(3.3)	0.947
the others n (%)	6(3.1)	4(4.4)	0.877
Maternal prenatal use of dexamethasone n (%)	77(40.31)	41(45.1)	0.545
Maternal prenatal use of antibiotics n (%)	2(1.0)	2(2.2)	0.838
DR chest compression n (%)	24(12.6)	24(26.4)	0.005
DR epinephrine n (%)	16(8.4)	10(10.8)	0.515
Apgar scores at 1 min median [Q1,Q3]	7(6, 9)	5(4, 8)	0.000
Apgar scores at 5 min median [Q1,Q3]	9(7, 9)	7(6, 9)	0.000
Pre-extubation			
PMA at extubation (wk) median [Q1,Q3]	30.6(29,31.8)	30(28,32)	0.153
Age at extubation (d) median [Q1,Q3]	8(4, 8)	9.1(5, 10)	0.000
Pulmonary surfactant treatment n (%)	163(85.3)	84(92.3)	0.000
Respiratory stimulant treatment n (%)	17(8.9)	22(24.2)	0.000
MV durations (d), median [Q1,Q3]	5(3.75, 7)	7(5, 10)	0.000
PH median [Q1,Q3]	7.4(7.38, 7.41)	7.34(7.29, 7.41)	0.000
PCO_2_ (mm Hg) median [Q1,Q3]	28(27, 30)	32(27.5, 36.8)	0.000
PO_2_ (mm Hg) median [Q1,Q3]	108(98.8, 125)	88(78, 101.8)	0.000
CRP (ng/dL) median [Q1,Q3]	0.05(0.01, 0.15)	0.05(0.01, 4.88)	0.001
RBC (10^12^/L) median [Q1,Q3]	4.29(4.1, 4.67)	3.91(3.38, 4.18)	0.000
Hb (g/L) median [Q1,Q3]	152.4(143, 162)	129.3(115, 144.5)	0.000
PLT (10^9^/L)median [Q1,Q3]	216.5(179,246)	210.1(167,239)	0.375
WBC (10^9^/L)median [Q1,Q3]	7.79(7.14,9.25)	8.13(7.12,11.06)	0.721
Respiratory rate (/min) median [Q1,Q3]	38.2(35, 40)	39.7(35, 43.75)	0.072
Heart rate (/min) median [Q1,Q3]	135(130, 140)	140(135, 145)	0.000
Highest FiO_2_ (%) median [Q1,Q3]	40(35, 50)	60(45, 68.75)	0.000
PIP (cm H_2_O) median [Q1,Q3]	14.8(14, 16)	15.7(14, 18)	0.003
FiO_2_ (%) median [Q1,Q3]	30(25, 30)	35(30, 35)	0.000
PEEP (cm H_2_O) median [Q1,Q3]	4.2(4, 5)	4.6(4, 5)	0.023
MV rate (/min) median [Q1,Q3]	30(25, 35)	35(30, 35)	0.000

### Predictive models for EF in BPD neonates

**Fig. 2:**
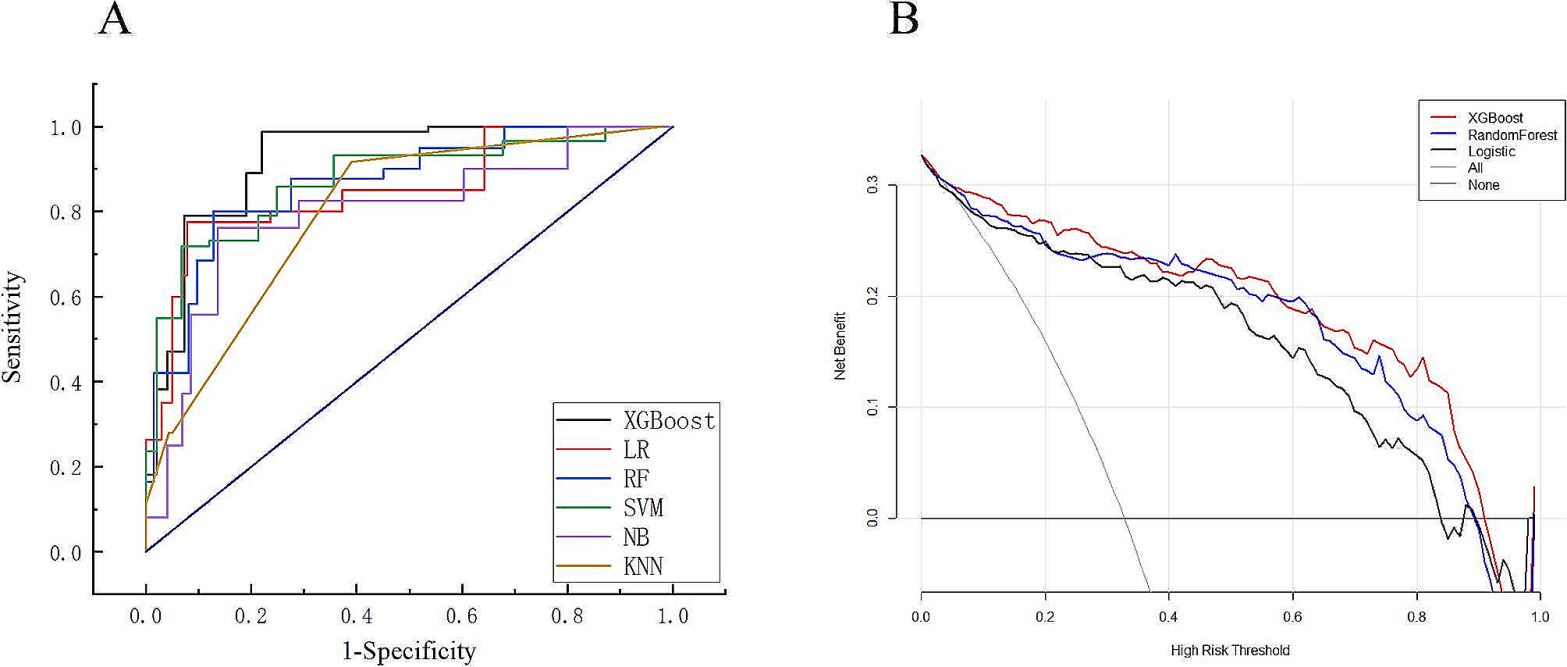
**(A)** Receiver operating characteristic (ROC) curves of six machine-learning models. The three best performing models were the XGBoost, RF, and LR models. XGBoost AUC = 0.873; RF AUC = 0.836; LR AUC = 0.787; NB AUC = 0.771; SVM AUC = 0.758; KNN AUC = 0.643. **(B)** Decision curve analysis (DCA) of the three prediction models. The net benefit curves for the three prognostic models are shown. X-axis indicates the threshold probability for critical care outcome, and Y-axis indicates the net benefit. Solid red line = XGboost model; solid black line = RF model; solid blue line = LR model. The preferred model is the XGboost model, the net benefit of which was larger over the range of the RF and LR models

**Fig. 3:**
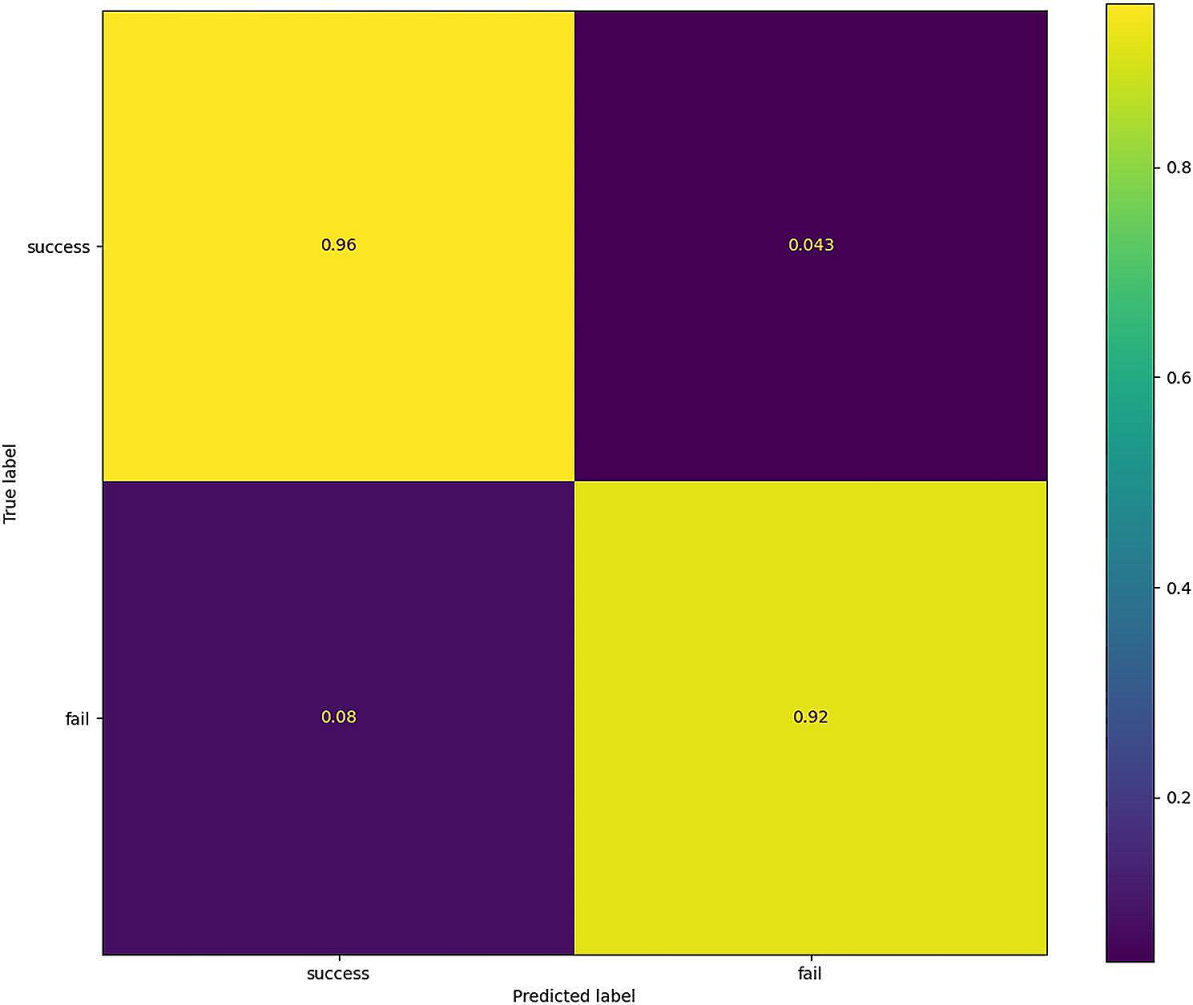
Confusion matrix of the XGBoost

**Fig. 4:**
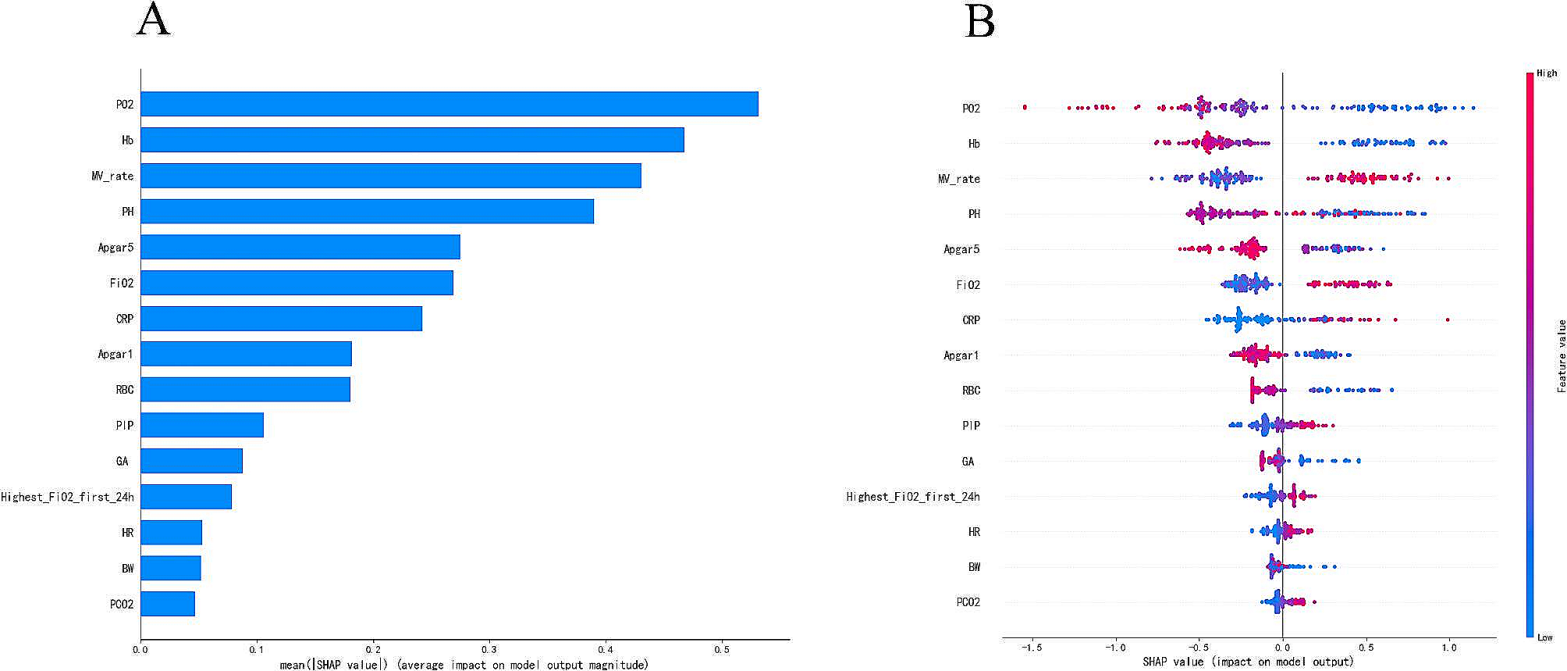
Top 15 features selected using XGBoost. **(A)** The corresponding variable importance score. X-axis indicates the importance score, which is the relative number of a variable that is used to distribute the data. Y-axis indicates the top 15 weighted variables. **(B)** The SHapley additive exPlanations (SHAP) framework for the features in the XGBoost model. Input variables are ranked in a descending order of feature importance. Red indicates positive contribution; blue indicates negative contribution

In imbalanced data, oversampling method with ADASYN algorithm was applied to achieve a balanced class distribution. In the development of the ML model, 23 key features were finally selected, including BW, GA, Apgar scores at 1 and 5 min, DR chest compression, the use of PS and respiratory stimulants, maternal disease (pregnancy-induced hypertension), highest FiO_2_ at the first 24 h, heart rate, MV rate, FiO_2_, PEEP, PIP, pH, pO_2_, pCO_2_, RBC, Hb, and CRP within 6 h prior to extubation, MV duration, and age at extubation. First, the AUC values of the six models (shown in Fig. 2A) were obtained, and the sensitivity and specificity analyses of these predictive methods are summarized in Table 2. The XGBoost, LR, and RF models showed the largest AUC values (XGBoost, AUC = 0.873; RF, AUC = 0.836; LR, AUC = 0.787). According to the DCA of the above mentioned three prediction models, the net benefit of the XGBoost model was larger than that of the LR and RF models, suggesting that the XGBoost model was optimal and the LR model was inferior (Fig. 2B). Confusion matrix showed the high performance of the best model (Fig. 3) and had superior results (Table 3). Figure 4A shows the feature importance analysis of the XGBoost model to intuitively reflect the importance of the features. The top 15 features were selected, and it turned out that three features, namely, pO_2_, Hb, and MV rate, were the most important predictors of EF. To obtain more complex correlations with EF, the SHAP values were calculated to show positive and negative effects. Visualization results are shown in Fig. 4B, with blue indicating a negative contribution and red indicating a positive contribution. Specifically, MV rate, FiO_2_, CRP, PIP, highest FiO_2_ in the first 24 h, heart rate, and pCO_2_ were positive predictors of EF, as increasing the values of these features increased the prediction of EF. In contrast, pO_2_, Hb, pH, Apgar score at 1 and 5 min, RBC, GA and BW were negatively correlated with EF, as smaller values of these features indicated better prediction of the model.

**Table 2:** Model performance in our study

	AUC (95%CI)	best cutoff	Youden index	sensitivity	specificity
XGboost	0.873(0.75–0.92)	0.668	0.727	0.896	0.838
RF	0.836 (0.71–0.85)	0.639	0.551	0.765	0.753
LR	0.787 (0.71–0.86)	0.627	0.531	0.745	0.749
NB	0.771 (0.69–0.82)	0.635	0.613	0.716	0.727
SVM	0.758 (0.65–0.80)	0.613	0.523	0.692	0.718
KNN	0.643 (0.59–0.69)	0.544	0.482	0.743	0.707

**Table 3:** The results of confusion matrix of XGBoost model

	accuracy	precision	recall	F1-score
XGboost	0.926	0.933	0.896	0.915

### Validation of predictive model of XGBoost

**Fig. 5:**
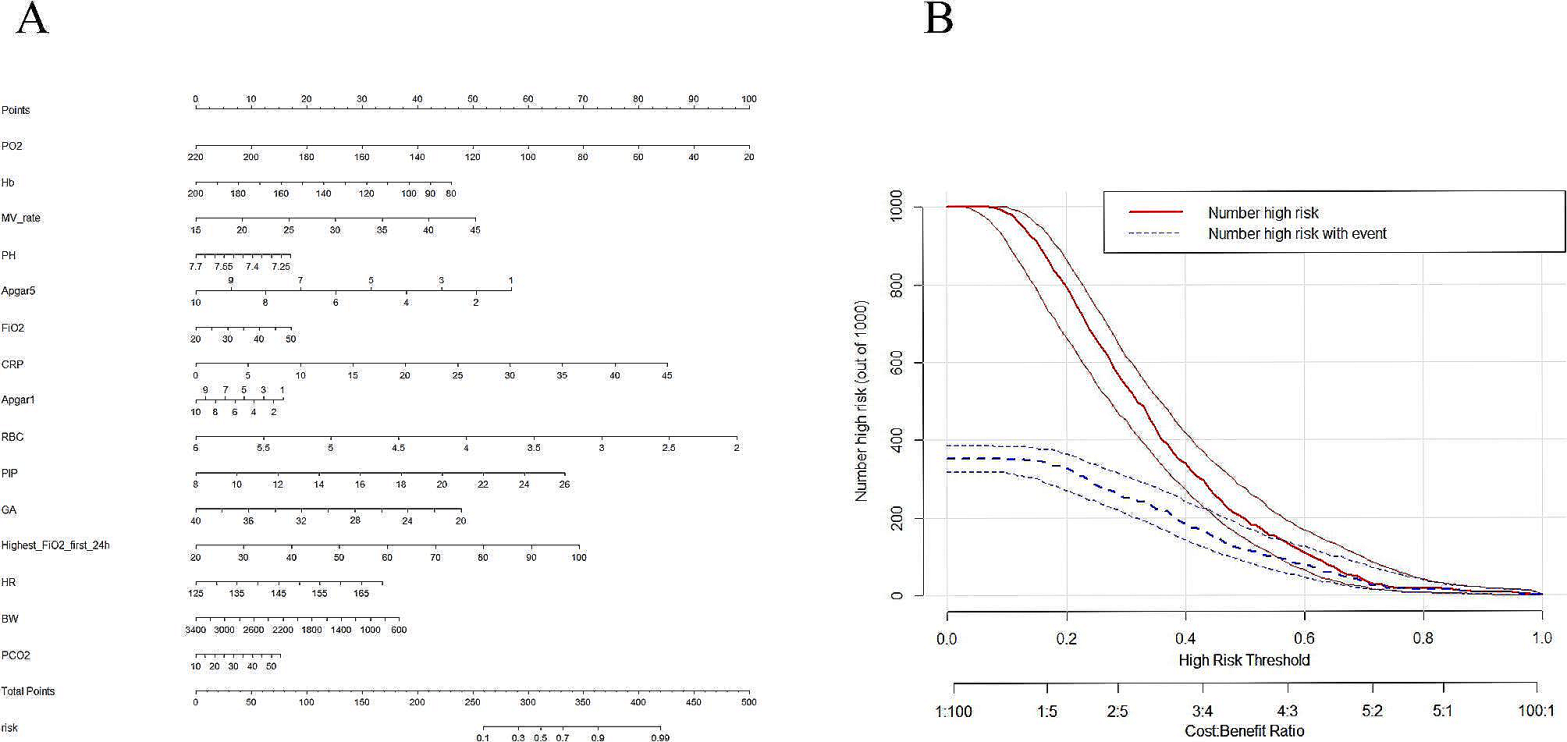
Risk nomogram and clinical impact curve (CIC) analysis of the XGBoost model. **(A)** Nomogram to estimate the risk of re-intubation. To use the nomogram, we first draw a line from each parameter value to the score axis for the score; the points for all of the parameters are then added; finally, a line from the total score axis is drawn to determine the risk of mortality on the lower line of the nomogram. **(B)** CIC analysis of the XGBoost model. The red curve (number of high-risk individuals) indicates the number of people who are classified as positive (high risk) by the model at each threshold probability; the blue curve (number of high-risk individuals with outcome) is the number of true positives at each threshold probability. CIC visually indicates that nomogram confers high clinical net benefit and confirms the clinical value of the XGboost model

To visualize the XGBoost model, the risk nomogram integrating 15 selected variables for the incidence of EF is shown in Fig. 5A. A CIC analysis was performed (Fig. 5B) to evaluate the clinical applicability of the risk prediction nomogram. The X-axis of the curve represents the number of patients or tests, and the Y-axis represents the number of true positives and false positives. The red curve (number of high-risk individuals) indicates the number of people who are classified as positive (high risk) by the model at each threshold probability. The blue curve (number of high-risk individuals with outcome) is the number of true positives at each threshold probability. In the CIC, when the threshold is greater than 0.65, the prediction model and the actual result are highly matched. The CIC visually showed that the nomogram had a superior overall net benefit within the wide and practical range of threshold probabilities and affected patient outcomes, indicating that the XGBoost model possessed significant predictive value.

## Discussion

In this study, the AUC values and DCA demonstrated that using the XGboost model could effectively predict EF in neonates. The pO_2_, Hb, MV rate, pH, Apgar score at 5 min, FiO_2_, C-reactive protein, Apgar score at 1 min, red blood cell count, PIP, GA, highest FiO_2_ at the first 24 h, heart rate, BW, pCO_2_ were good predictors of EF in neonates. To our knowledge, this is the first mathematical model to predict EF in neonates with BPD.

XGBoost, a decision tree–based algorithm, was found to be a better algorithm for ML and prediction [24]. There is growing evidence that XGBoost-based ML algorithm is a competitive alternative to traditional regression analysis, and it is used in predicting clinical adverse outcomes due to its excellent precision value and performance [25–28]. In the present study, we compared the predictive efficiency of different models and found that the XGBoost model demonstrated advantages over other models, including the LR and RF models.

The definition of EF has been quite variable among previous studies, ranging from 2 to 7 days after extubation [3]. A shorter period (prior to 48 h) may better distinguish between re-intubation for pulmonary insufficiency and re-intubation for other reasons such as sepsis or surgery [29]. Conversely, a longer period (7 days) accounts for a greater proportion of EF [30]. In this study, we used a time frame ≤ 72 h to define EF, and the results indicated that EF could be predicted effectively.

In the present study, 23 variables were evaluated to improve the usability of our model, and eventually 15 key features were selected, including pO_2_, Hb, MV rate, pH, Apgar score at 5 min, FiO_2_, C-reactive protein, Apgar score at 1 min, red blood cell count, PIP, GA, highest FiO_2_ at the first 24 h, heart rate, BW, pCO_2_. Previous studies have indicated that lower GA, BW, and Apgar score were important factors associated with an increased risk of EF [7, 8, 10, 31], which is consistent with the present study. Lower GA, BW, and Apgar score usually indicate underdeveloped lungs, low lung compliance, high airway resistance, and an immature central respiratory drive. A high FiO_2_ requirement on day 1 of life could also be a marker of the severity of respiratory distress syndrome and brain immaturity [6]. In our study, the highest FiO_2_ during the first 24 h of life was a significant predictor of EF.

We speculated that pH, pO_2_, and pCO_2_ within 6 h before extubation were significantly predictive of extubation readiness. Lower pH and pO_2_ and higher pCO_2_ indicate hypoventilation or severe pulmonary disease [2, 32], and are remarkable predictive factors for EF according to their SHAP values. In addition, abnormal vital signs such as heart rate were associated with a higher EF risk. The basic factor is commonly used in clinical practice, representing the vital status of a patient, and is included in many prediction models [33, 34].

As expected, pre-extubation Hb, RBC, and CRP levels were significantly different between the two groups. It remains controversial whether anemia causes apnea in premature neonates [25–27, 35], and this issue has not been well-studied in full-term newborns. Understandably, patients may be affected if the major oxygen transporter (erythrocyte and Hb) is reduced to a certain level. The association between CRP and EF could be explained by the fact that CRP is a measure of infection in the lungs, and, as shown in Fig. 3B, a higher CRP level was related to a higher EF risk.

The current study also showed that MV parameters before extubation, such as MV rate, FiO_2_, PIP, contribute to EF prediction in neonates. Previous studies have explored the benefits of MV parameters prior to extubation [25–28]. These studies indicated that there is a higher risk of EF when the effort of breathing results in a lower spontaneous tidal volume and there is an increased load on the respiratory muscles, as indicated by a high PIP, MV rate, and FiO_2_. In addition, when the patients were extubated from a high level of ventilator support, as shown by higher FiO_2_, PIP, and MV rates, their ability to sustain spontaneous breathing after extubation was overestimated. Therefore, the results of the present study provide support for serious consideration of EF in neonates prior to extubation.

This study has several limitations. First, there is disagreement regarding the definition of EF. The definition adopted in the present study was a time frame ≤ 72 h prior to re-intubation. However, a shorter period may miss a proportion of EF. Second, the data was from a single center, and there was no additional data to validate the robustness and universality of the model. Thus, further multicenter prospective study with larger samples should be conducted to validate the prediction model.

In conclusion, the current study screened 15 key features associated with EF and developed an XGBoost model that can better predict EF than other predictive models in neonates diagnosed with BPD.

## Data Availability

The authors confirm that data supporting the findings of this study are available within the article.
